# In Vitro Genotoxicity Assessment of Functional Ingredients: Betaine, Choline, and Taurine

**DOI:** 10.3390/foods10020339

**Published:** 2021-02-05

**Authors:** Julen Sanz-Serrano, Ariane Vettorazzi, Damian Muruzabal, Amaya Azqueta, Adela López de Cerain

**Affiliations:** 1 Department of Pharmacology and Toxicology, School of Pharmacy and Nutrition, Universidad de Navarra, Irunlarrea 1, 31008 Pamplona, Spain; jsanz.3@alumni.unav.es (J.S.-S.); avettora@unav.es (A.V.); dmuruzabal@alumni.unav.es (D.M.); acerain@unav.es (A.L.d.C.); 2 Navarra Institute for Health Research, IdiSNA, Irunlarrea 3, 31008 Pamplona, Spain

**Keywords:** mutagenicity, genotoxicity, functional ingredients, betaine, choline, taurine, Ames test, comet assay, micronucleus test

## Abstract

This article focuses on a complete in vitro genotoxicity assessment of three nutrients widely used as functional ingredients in the European market: betaine, choline, and taurine. The European Food Safety Authority (EFSA) tiered approach for food additives in concordance with the safety assessment of chemicals in food developed by Food and Agriculture Organization/World Health Organization (FAO/WHO) was followed; the miniaturized Ames test in *Salmonella typhimurium* TA97a, TA98, TA100, TA102, and TA1535 strains (following the principles of Organization for Economic Co-operation and Development (OECD) 471), and the micronucleus test (OECD 487) in TK6 cells were performed. In addition, the in vitro standard and enzyme-modified (human 8-oxoguanine DNA glycosylase 1 (hOGG), endonuclease III (EndoIII), human alkyladenine DNA glycosylase (hAAG)) comet assay (S9−/S9+) was conducted in order to assess the potential premutagenic lesions in TK6 cells. None of the compounds produced any signs of genotoxicity in any of the conditions tested. This article increases the limited evidence available and complements the EFSA recommendations for the in vitro genotoxicity testing of nutrients.

## 1. Introduction

Over the past decade, new foods have emerged containing intentionally added compounds with a specific beneficial effect on health beyond their food counterparts. Although there is a lack of global consensus on its definition yet, these so-called functional foods aim to maintain a physiological function, to prevent a disease, and/or to promote well-being, beyond their basic nutritional function [1].

Betaine, choline, and taurine are three non-essential nutrients naturally present in food, but they can also be intentionally added to food [2,3,4]. These compounds are part of many vital functions in the body and have been related with different health effects. Indeed, several health claims related with these nutrients have been submitted for evaluation under the EU Nutrition and Health Claims legislation (Table 1) [5].

The safety assessment of functional foods might fall under three regulations, “novel food” [13], “fortified foods” [14], or also in “foods for specific groups” such as in infant formula or diet replacement foods [3]. The EU general food law [15] and the above-mentioned regulations specify that the safety should be guaranteed. Although the responsibility for the safety of these foods lies with the food business operator, the addition of any substance in food is regulated in the European Union (EU) by the establishment of positive lists annexed to the above-mentioned regulations.

In the case that a new substance is proposed by a food business operator as a “novel food” or for use as a “source of nutrients” in food supplements, foods for the general population, or foods for specific groups, the European Food Safety Authority (EFSA) is requested by the European Commission to perform an assessment of the safety and of the bioavailability of a nutrient from the proposed source [16]. In general, the toxicity testing follows the same tiered approach as for food additives in concordance with the safety assessment of chemicals in food developed by Food and Agriculture Organization/World Health Organization (FAO/WHO) [17,18].

Regarding betaine, addition to beverages, cereal products, confectionary, or dairy products in the EU was firstly intended fifteen years ago, but the available data were not sufficient to rule out the safety concerns (Table 2) [19]. Nonetheless, recent scientific opinions of the European Food Safety Authority (EFSA) expressed no safety concerns to the addition of betaine in novel foods for intense muscular effort, foods for sportsmen, weight control, and special medical purposes, under determined conditions (Table 2) [2,20]. This has led to its inclusion in the positive list of authorized novel foods [21]. Choline, under its different forms, as well as taurine, are authorized for their addition in infant formula and follow-on formula, food for special medical purposes, and total diet replacement for weight control [3]. In addition, choline is also authorized to be added in processed cereal-based food and baby food [3]. Nonetheless, the only safety assessment of choline under European regulation is included in the “Safety for consumers” section of the safety assessment as a feed additive for all animal species (Table 2). In the case of taurine, its main market as a novel food ingredient lies in “energy” or sport drinks [22]. In fact, the ingestion of taurine at the levels present in these drinks, although with a need of more exposure data, was established as “Not of safety concern” by the EFSA in 2009 (Table 2) [23].

One of the cornerstones of safety assessment strategies is the evaluation of genotoxicity, which includes an in vitro first phase composed by a mutagenicity assay that detects punctual gene mutations, commonly the bacterial reverse mutation assay (Ames) test, and a test that assesses DNA damage at the chromosomal level [17,18]. All these methods should follow the internationally agreed test guidelines described by the Organization for Economic Co-operation and Development (OECD) and be performed on the basis of Good Laboratory Practice (GLP) [17,18]. Nonetheless, the available genotoxicity data for these functional ingredients (Table 2) is not fully in line with the current toxicity testing approach recommended by EFSA [18]. Additionally, these strategies include assays that only detect mutations, so they do not provide information about the potential premutagenic lesions and thus the mechanism of action of the compounds.

This work focuses on a complete in vitro genotoxicity assessment of betaine, choline, and taurine, based on the existing safety strategies applicable to food and feed safety assessment [17,18], including the miniaturized Ames test (following the principles of OECD 471) and the in vitro micronucleus (MN) assay (OECD 487), and the in vitro standard and enzyme-modified comet assay.

## 2. Materials and Methods

### 2.1. Chemicals and Reagents

All the chemicals were purchased from Sigma Aldrich (Steinheim, Germany) unless otherwise stated below. RPMI 1640 medium (‘ATCC modified’), and heat-inactivated fetal bovine serum (FBS) were obtained from Gibco (Brooklyn, NY, USA); Dulbecco’s Phosphate-Buffered Saline (DPBS) was obtained from Gibco (Paisley, UK); Phosphate-Buffered Saline (PBS) tablets were obtained from Oxoid (Hampshire, UK); Penicillin–streptomycin was acquired from Lonza (Cologne, Germany); Mutazime S9 preparation from livers of Aroclor 1254-induced rats, S9 fraction from livers of Aroclor 1254-induced rats and mitomycin C (MitC) were purchased from Moltox (Boone, NC, USA); Cell sorting set-up beads for blue lasers (beads) and Sytox dye were obtained from Invitrogen (Eugene, OR, USA); RNAase and 0.4% Trypan Blue were obtained from Invitrogen (Carlsbad, CA, USA); enzymes 3-alkyladenine DNA glycosylase (hAAG) and endonuclease III (Endo III) were obtained from New England Biolabs (Ipswich, MA, USA), and Human 8-oxoguanine DNA glycosylase 1 (hOGG-1) was obtained from R&D Systems (Minneapolis, MN, USA).

### 2.2. Cell Culture

TK6 cell line (human-derived lymphoblastoid cells) was obtained from the American Type Culture Collection (ATCC) (Manassas, VA, USA). Cells were maintained in RPMI 1640 medium (supplemented with 10% heat-inactivated FBS, 100 U/mL penicillin, and 0.1 mg/mL streptomycin) as a suspension culture (0.2–1 × 10^6^ cells/mL) in continuous agitation in a humidified incubator at 37 °C and 5% CO_2_ for no longer than 60 days. Cell doubling time was approximately 15 h during the experimental period.

### 2.3. Miniaturized Ames Test

Betaine (CAS no. 107-43-7), choline (CAS no. 67-48-1), and taurine (CAS no. 107-35-7) were tested in five *S. typhimurium* strains, TA97a, TA98, TA100, TA102, and TA1535 (Moltox, NC, USA), with (S9+) and without (S9−) metabolic activation, using the 6-well-plate incorporation methodology described by Burke et al. with some modifications [29]. The assay was conducted according to the principles of the OECD guideline 471 for the Ames test [30].

The compounds were tested at five concentrations (1/3 dilutions) in the following ranges: 21–5000 µg/well for betaine and choline, and 7–1667 µg/well for taurine, due to solubility issues. Lower concentrations of betaine (0.09–21 µg/well) were tested in TA98 strain (S9+) due to toxicity. Positive controls were included in each experiment: 2 μg/well 2-aminoanthracene (AA) for TA97a and TA1535, 10 μg/well 2-aminofluorene (AF) for TA98 and TA100, and 20 μg/well AF for TA102, in the absence of S9; and 10 μg/well 4-nitro-o-phenylenediamine (NPD) for TA97a, 20 μg/well NPD for TA98 and TA100, 0.625 μg/well MitC for TA102, and 2 μg/well sodium azide (NAAZ) for TA1535, in the presence of S9. AA, AF, and NPD were dissolved in DMSO, and MitC and NAAZ were dissolved in water.

The following criteria were used to consider a compound to induce point mutations: (a) a two-fold increase at one or more concentrations in the number of revertant colonies per plate in at least one strain with or without metabolic activation system, and (b) a concentration-related increase over the range tested.

Briefly, tubes placed in a PHMT-PSC24 Thermo shaker (Grant Bio) at 47 °C and 300 rpm were filled in the following order: 20 µL of test compound solution, positive control or solvent (i.e., H_2_O), 500 µL of histidine–biotin top agar (9.5 µg/mL histidine, 11.1 µg/mL biotin, 4.5 mg/mL sodium chloride, and 5.5 mg/mL Bacto agar, in distilled water), 25 µL of 2 × 10^9^ bacteria/mL, and 100 µL of PBS or S9 preparation (10% Mutazime S9). Then, each tube was poured onto minimal-medium-agar-filled wells in 6-well plates. Each condition was studied per triplicate in three separated wells. Plates were incubated at 37 °C and saturated humidity for 48–72 h until revertant colonies were counted by visual examination.

### 2.4. In Vitro MN Test

Betaine, choline, and taurine were tested for MN induction in TK6 cells following the recommendations of OECD guideline 487 [31]. Two independent experiments were performed for each compound.

The compounds were tested at four concentrations (1/3 dilutions) according to the limit established by OECD guideline 487 (i.e., 74–2000 µg/mL) for 3 h (S9−/S9+) and 24 h (S9−) [31]. For the metabolic activation of the compounds, the final concentration of S9 was 1% S9 fraction with 1.5 mg/mL β-nicotinamide adenine dinucleotide phosphate sodium salt hydrate (NADP) and 2.7 mg/mL DL-isocitric acid as cofactors. Positive controls were included in each experiment: 4 µg/mL of cyclophosphamide (CP) for 3 h treatment in the presence of S9 and 10 ng/mL colchicine (COL) for 24 h treatment.

The following criteria was used to consider a compound to induce MN: (a) a three-fold increase in MN at one or more concentrations in at least the short treatment (S9−/S9+) or long treatment (S9−), and (b) a concentration-related increase over the range tested.

TK6 cells were seeded in a 12-well plate (6 × 10^5^ cells/1 mL for 3 h of treatment and 3 × 10^5^ cells/1 mL for 24 h of treatment) treated with the test compound, positive controls, or solvent (i.e., H_2_O) for either 3 h (S9−/S9+) or 24 h (S9−), and incubated for 1.5–2 cell cycles (since the beginning of the treatment). Then, cells were centrifuged (141× *g*, 8 min, 4 °C), resuspended in cold 0.025 mg/mL Ethidium Monoazide Bromide (EMA) solution (in 2% FBS/DPBS) and incubated for 20 min in ice under 60 W direct light (30 cm). After the incubation, cells were washed with 2% FBS/DPBS by centrifugation (141× *g*, 8 min, 4 °C) and incubated for 1 h in lysis solution 1 (0.2 µM Sytox dye, 1 mg/mL RNAase, 0.584 mg/mL sodium chloride, 1 mg/mL trisodium citrate dihydrate, 0.3 µL/mL IGEPAL). Then, lysis solution 2 (0.2 µM Sytox dye, 1.5 µL/mL beads, 85.6 mg/mL sucrose, 15 mg/mL citric acid) was added, and cells were incubated for additional 30 min. Both lysis incubations were performed at room temperature in darkness. Samples were stored at 4 °C for no more than 24 h until the analysis in a cytometer FACSCanto^TM^ II Six Colors (BD, Franklin Lakes, NJ, USA) was performed. At least 20,000 healthy nucleated cells were scored per condition, and MN were determined using FlowJo^TM^ V10.2 software from BD (Franklin Lakes, NJ, USA) by following the MicroFlow Instructions Manual (Litron Laboratories, Brighton, NY, USA).

In order to evaluate the cytotoxicity, a fixed number of beads were added (included in lysis solution 2) to each condition and were counted during the cytometry analysis. The relative survival value (RS) for each condition was obtained dividing each healthy nuclei/bead ratio by the one obtained in the vehicle control.

### 2.5. Enzyme-Modified Comet Assay

Betaine, choline, and taurine were evaluated by the enzyme-modified comet assay in TK6 cells. The assay was carried out using the enzyme-modified 12 minigels/slide format as described by Muruzabal et al. [32]. The following three enzymes were used in combination with the standard alkaline comet assay: hOGG-1 for the detection of oxidized purines (mainly 8-oxoguanine) [33], Endo III for oxidized pyrimidines [34], and hAAG for alkylated lesions [35]. Three independent experiments were performed for each compound.

The compounds were tested for 3 h at five concentrations in a range of 25–2000 µg/mL (S9−/S9+). The final concentration of S9 was 1% S9 fraction with 1.5 mg/mL NADP and 2.7 mg/mL DL-isocitric acid as cofactors. Methyl methanesulphonate (MMS) at 20 µM was included as positive control in each experiment.

The following criteria was used to consider a compound to induce strand breaks, oxidized or alkylated bases: (a) a statistically significant increase in percentage tail intensity at one or more concentrations (S9−/S9+), (b) a concentration-related increase over the range tested.

TK6 cells were seeded (6 × 10^5^ cells in 1 mL) in a 12-well plate and treated with the test compound, positive control, or solvent (i.e., H_2_O). Briefly, cells were washed by centrifugation (250× *g*, 5 min) after the treatment, mixed with low melting point agarose, and 10 minigels (5 µL, 0.82% agarose) were placed on agarose-precoated slides. Once the gels were solidified, the slides were immersed for 1 h in lysis solution. Then, the slides were washed in cold enzyme reaction buffer three times for 5 min each and placed on a cold 12-Gel Comet Assay Unit™. Each of the following solutions were added per couple of gels (30 µL/gel): lysis buffer (without Triton X-100), enzyme reaction buffer, and two more with each of the enzymes 5–10 U/mL hAAG, 33.3 U/mL Endo III, or 3.3 U/mL hOGG-1. The 12-Gel Comet Assay Units™ were placed in the incubator at 37 °C for 1 h in a moist box. After that, slides were immersed for 40 min in alkaline solution, and then, electrophoresis was carried out at 1.2 V/cm for 20 min in the same buffer. After the electrophoresis, slides were immersed in DPBS and water (10 min each), and then dried by using 70% and 96% ethanol (15 min each). Comets were stained by adding a drop of 1 µg/mL of 4,6-diamidino-2-phenylindole (DAPI) on top of each gel, covered with a coverslip, and incubated for at least 30 min. Semi-automatic software Comet Assay IV from Instem (Staffordshire, UK) was used to randomly score 50 comets per gel (100/condition) under fluorescence microscopy (Eclipse 50i Nikon). The percentage of DNA in tail was used as the comet descriptor. Gels immersed in lysis buffer during enzyme incubation time were used for the measurements of strand breaks (SBs) and alkali labile sites (ALS). Net enzyme-sensitive sites were calculated by subtracting the percentage DNA in tail obtained after reaction buffer incubation from that obtained after the enzyme incubation.

### 2.6. Proliferation Assay

The cytotoxicity of the three compounds was evaluated by assessing their impact on cell proliferation. After the treatment (see Section 2.5. Enzyme-modified comet assay), cells were washed by centrifugation (250× *g*, 5 min) with DPBS and reseeded in fresh medium. The number of cells in each condition were counted twice right after the treatment, and after 24 h and 48 h of incubation in fresh medium. Cell were counted after 24 h to adjust the concentration of cells if needed. In all cases, Countess™ Automated Cell Counter (Invitrogen, CA, USA) and Trypan Blue were used. Total suspension growth (TSG) was calculated dividing the number of cells after 48 h by the number of cells before the treatment. Relative suspension growth (RSG) was calculated as a percentage by dividing the TSG from each condition tested by the TSG of the solvent control.

### 2.7. Statistical Analysis

Miniaturized Ames test results are shown as the mean and SD for the technical triplicates of each condition.

The mean and SD values of MN/1000-nuclei ratio for the duplicate independent experiments were calculated.

The median values of DNA in the tail of the 100 comets scored per condition were calculated, and the mean and SD values for the triplicate experiments are shown. The mean DNA in the tail for each condition was compared with the vehicle control value using the Kruskal–Wallis test followed, if needed, by the post-hoc Bonferroni test. Statistical significance was set at *p* > 0.05 in both tests. The Statistics and Data Analysis (STATA) software v12.1 (College Station, TX, USA) was used.

In the proliferation assay, the mean and SD for RSG of the triplicate experiments are shown.

## 3. Results

### 3.1. Miniaturized Ames Test

Table 3 presents the results as the mean and SD of the technical triplicates in one independent experiment. Betaine, choline, and taurine did not revert the mutation in S. typhimurium strains (S9−/S9+); none of the conditions tested showed twice the control values and a dose–response increase. The positive control chemicals produced the expected increase in mutation frequency, and the bacterial phenotypes were confirmed.

Betaine induced toxicity in TA98 (S9+) at the concentrations from 62 µg/well to 5000 µg/well. Revertant colony induction was not observed in an additional experiment conducted at lower doses (0.03–21 µg/well) (data not shown). This section may be divided by subheadings. It should provide a concise and precise description of the experimental results, their interpretation, as well as the experimental conclusions that can be drawn.

### 3.2. In Vitro MN Test

Table 4 shows the mean ± min/max values of MN/1000-nuclei ratio and relative survival (RS) for the duplicate experiments together with the individual experimental values. None of the compounds induced a concentration-related three-fold increase in MN in TK6 cells compared to the vehicle control in any of the conditions tested. Cells treated with CP (S9+) and COL showed a significant increase in MN as compared with the vehicle control cultures.

### 3.3. Enzyme-Modified Comet Assay and Proliferation Assay

**Figure 1:**
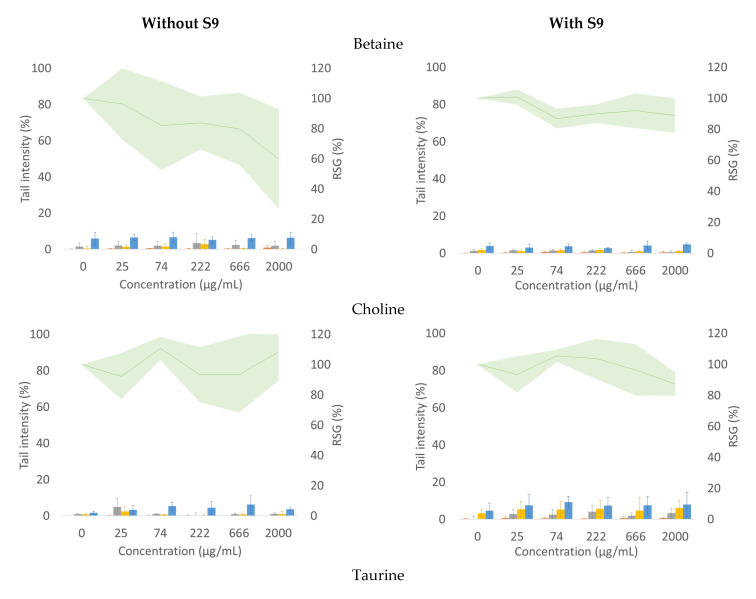

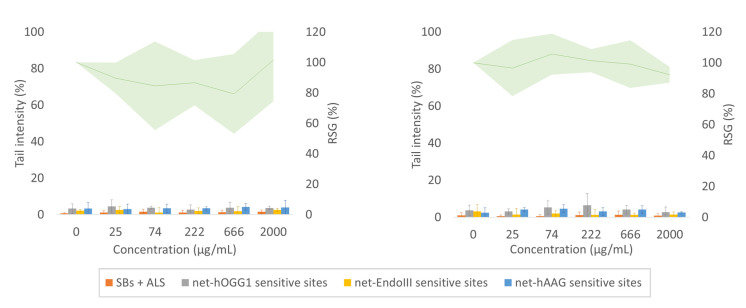
Enzyme-modified comet assay (bars) and proliferation assay (line) for betaine, choline, and taurine in TK6 cells after 3 h treatment with (right column) and without (left column) external metabolic activation (S9). Mean percentage of tail intensity and relative suspension growth (RSG) are shown for triplicate experiments. Error bars in tail intensity and pale area in RSG stand for SD.

Figure 1 shows the DNA tail intensity (%) and cell proliferation (RSG) of triplicate experiments for each condition. None of the three compounds showed statistically significant induction in DNA strand breaks (SB) or enzyme-sensitive sites (oxidized and alkylated bases) compared to vehicle control values. Betaine seemed to impair cell proliferation (RSG < 80%) without metabolic activation (Figure 1). Positive controls showed the expected percentage in tail intensity of net-enzyme sensitive sites; the mean ± SD of all the independent experiments were as follows: 25.0 ± 16% for hOGG1, 21.0 ± 5.0% for Endo III, and 83.9 ± 8.0% for hAAG.

## 4. Discussion

Betaine, choline, and taurine were evaluated following the principles of the genotoxicity testing strategy proposed by EFSA [18]. However, the Ames test was performed in a miniaturized version using 6-well plates, although taking into account the OECD 471. The in vitro MN test was carried out following the OECD 487. In order to provide more mechanistic information, the in vitro comet assay was also performed in its standard version and in combination with the enzymes hOGG, EndoIII, and hAAG to evaluate the potential induction of premutagenic lesions.

There are currently few articles assessing the genotoxicity of these functional ingredients. In the late 1980s, Hoorn et al. were the first investigators assessing the mutagenicity of betaine [36]; no mutagenic response was observed in the Ames test (strain TA100) treated with concentrations ranging from 1 to 10,000 µg/well (S9−/S9+). More recently, the EFSA Scientific Panel on Dietetic Products, Nutrition and Allergies (NDA) assessed the in vitro genotoxicity of betaine by the Ames test and the chromosome aberration test (CA) [19]. Concentrations up to 5000 μg/plate produced no mutagenicity in *S. typhimurium* strains TA1535, TA1537, TA1538, TA98, and TA100 (S9−/S9+), and concentrations up to 10 mg/mL were not clastogenic in human lymphocytes (S9−/S9+). The NDA panel referred no concerns with respect to genotoxicity of betaine, although the genotoxicity testing strategy was not fully in line with current EFSA recommendations [20]. The results in the current article provide additional evidence that betaine is not mutagenic in *Salmonella typhimurium* strains TA1535, TA97a, TA98, TA100, and TA102, nor clastogenic or aneugenic in TK6 cells, and it did not induce either DNA breakage, ALS, oxidized bases, or alkylated bases in TK6 cells. The miniaturized Ames test and the in vitro MN test complete the EFSA conclusions regarding the in vitro genotoxicity of betaine. The standard and enzyme-modified comet assay also support and complement the available evidence about the absence of genotoxicity of betaine.

In a Screening Information Dataset (SIDS) of choline chloride [26], the OECD referred to four articles in which the Ames test was performed, but only two full articles were found in the available literature [37,38]. Two studies tested up to 10 and 20.83 mg/plate in *S. typhimurium* strains TA98, TA100, TA1535, and TA1537 [26,38], whilst the other two also included TA1538 and *E. coli* WP2 uvrA, with choline doses up to 5% (*v*/*v*) (specific concentration not reported) and 5 mg/plate (S9−/S9+), respectively [26,37]. Moreover, the SIDS report included a bacterial gene mutation assay in E. coli using concentrations up to 70 mg/mL (S9−) [39] and a gene conversion assay in Saccharomyces cerevisiae D4 using concentrations up to 50 mg/mL (S9−/S9+) [37]. No signs of mutagenicity were observed in any of the assays. The SIDS included the following three articles that assessed genotoxicity at a chromosomal level [26], although only one full article was found in the available literature [40]; no clastogenic activity was detected in CHO cells, applying CA in concentrations up to 0.5 mg/mL and 5 mg/mL in two parallel studies, and no increase in the incidence of SCE was observed up to 5 mg/mL. In the current article, the results in the miniaturized Ames test, MN test, and the standard and enzyme-modified comet assay agree with the available evidence and support the OECD conclusion that choline chloride does not have any genotoxic potential [26].

In 1999, the SCF firstly evaluated the safety of the constituents of “energy” drinks, including taurine, among others, and concluded that taurine had no genotoxic, teratogenic, or carcinogenic potential [27]. Only two of the three articles that EFSA included in the evaluation of the in vitro genotoxicity of taurine were found in the available literature [24]; a single concentration of 125.15 µg/mL of taurine was not able to induce chromosomal damage assessed by SCE and CA in CHO cells [41]. Yamada et al. did not observe mutagenicity in a Rec-assay using B. subtilis HI7 and M45 strains up to 10 mg/disc [42]. The more recent reports of EFSA, in 2003 and 2009, did not add any new studies to this conclusion [23,28]. After the latest version of the EFSA opinion on “energy” drinks, Turkez et al. tested 25, 50, and 100 µg/mL of taurine in human lymphocyte cultured cells for 72 h by SCE and micronucleus test [43]; no chromosomal damage was observed at any of the conditions tested. In this regard, the present article agrees with the available evidence and contributes to the safety strategy recommended by the EFSA. The comet assay showed that taurine did not produce any oxidized lesions in combination with the enzymes EndoIII and hOGG1, although some antioxidants have been reported as potentially damaging pro-oxidants at higher concentrations [44,45]. In this context, taurine did not produce either DNA breakage, ALS, or alkylated lesions.

It is to mention that all the test concentrations recommended by the OECD guidelines and thus, those tested in this article, are much higher than the naturally occurring in the human body. Betaine levels in blood are maintained in a narrow range within 2.3–7.0 µg/mL [46], but some studies reported an increase of 19.8 ± 11.7 µg/mL and 15.5 ± 6.2 in subjects who received 4 g/day and 3 g/day of betaine, respectively, during 12 weeks [20,47]. Fasting plasma-free choline mean levels range from 0.7 to 2.1 µg/mL [48], and taurine plasma concentration in humans ranges 5.5 ± 1.0 µg/mL, whilst whole blood taurine concentration is 28.4 ± 4.4 µg/mL [49]. Taurine plasma levels can peak to 12.8 ± 1.6 µg/mL after 400 mg/day for 7 days [49]. Taking into account the results in this article and the available literature, it seems that no genotoxicity could be expected from the average ingestion of these compounds.

## 5. Conclusions

The functional ingredients betaine, choline, and taurine were not mutagenic nor genotoxic in the miniaturized Ames test, MN test and, standard and enzyme-modified comet assay, in any of the conditions tested. This assessment was based on the existing safety strategies for additives and other chemicals present in food [15]. The results agree and complement the scarce evidence available, helping implement the way toward a better safety assessment for functional foods.

## Figures and Tables

**Table 1:** Health claims evaluated under the European Union regulation of betaine, choline, and taurine *.

Nutrient	Status	Health Relationship		EFSA Opinion Reference	Commission Regulation (EU)
Betaine	Authorized	Contribution to normal homocysteine metabolism	>500 mg/portion or 1.5 g/day >4 g/day increase cholesterolemia	[6]	[7]
Choline	Authorized	Contribution to normal homocysteine metabolism	Food which contains at least 82.5 mg/100 g or 100 mL or per single portion of food	[8]	[7]
		Contribution to normal lipid metabolism			
		Maintenance of normal liver function			
	Non-authorized	Contribution to normal cognitive function		[8]	
		Maintenance of normal neurological function			
		Development of brain of infants and young children (0–3 years)		[9]	[10]
Taurine	Non-authorized	Delay in the onset of fatigue and enhancement of physical performance		[11]	
		Protection of DNA, proteins and lipids from oxidative damage			
		Energy-yielding metabolism			
		Contribution to normal cognitive function		[12]	
		Immune system protection			
		Delay in the onset of physical fatigue during exercise			
		Maintenance of normal cardiac function			
		Supports “metabolism processes”			
		Maintenance of normal muscle function			

* EU Register of nutrition and health claims made on foods (v.3.5) (accessed on 21 December 2020) [5].

**Table 2:** Summary on the safety for consumers of betaine, choline, and taurine as food ingredients or supplements assessed at the EU level and the in vitro genotoxicity assessment carried out.

	Reference	Regulatory Context	Aim	Conclusion	In Vitro Genotoxicity Assessment
Betaine	EFSA Journal 2005; 191, 1–17 [19].	Use as a novel food ingredient in the context of Regulation (EC) No 258/97 *.	In beverages, cereal product, confectionary and dairy product. Proposed intake of 4 g/day.	Some safety concerns remained unresolved; safety for the intended use not established.	(−) AT, (−) CA.
	EFSA Journal 2017; 15(11): 5057 [20].	Safety as a novel food pursuant to Regulation (EC) No 258/97 *.	Foods for intense muscular effort. Proposed intake of 2.5 g/day or 1.25 g/serving.	Safe at maximum intake of 400 mg/day (6 mg/kg bw/day for sportspeople > 10 years) in addition to the background exposure.	No genotoxicity concerns based on EFSA, 2005.
	EFSA Journal 2019; 17(4): 5658 [2].	Safety as a novel food pursuant to Regulation (EU) 2015/2283.	Foods for sportspeople (>10 years), weight control, and special medical purposes. Max use level ranged from 20–500 mg/100 g.	New conditions are safe (if <400 mg/day including supplements), except for medical purposes for <18 years	-
Choline	EFSA Journal 2011; 9(9): 2353 [24].	Authorization of additives for use in animal nutrition in the context of Regulation (EC) No 1831/2003.	Safety of choline for the target species, consumers, users and environment, and its efficacy.	No UL has been established in Europe to date. Refers to IOM report of a UL of 3500 mg/person/day for adults [25].	No genotoxicity concerns [26]: (−) AT (3x), (+) CA, (−) CA (2x), (+) SCE, (−) SCE (2x), (−) GCA.
Taurine	SCF, 1999 [27]	Inclusion as constituents of “energy” drinks.	Safety of taurine as constituent of “energy drinks.	Insufficient available data.	No genotoxic potential **: (−) SCE, (−) CA, (−) RA.
	SCF, 2003 [28].	Inclusion as constituents of “energy” drinks.	Safety of taurine as constituent of “energy” drinks.	Concerns related with behavioral effects and impaired motor performance at the lowest dose tested (300 mg/kg/day bw in rats); more neurological studies are needed.	No genotoxicity concerns based on SCF, 1999.
	EFSA Journal 2009; 935, 1–31 [23].	Use as constituents of “energy” drinks in the context of Regulation (EC) 178/2002.	Safety of taurine as individual ingredient of “energy” drinks.	The exposure to taurine at the levels presently used in “energy” drinks is not of safety concern, but more exposure data are needed.	No new studies available. No genotoxicity concerns based on SCF, 1999.

Abbreviations: AI: Adequate intakes; AT: Ames test; CA: Chromosomal aberration test; DRV: Dietary reference values; EFSA: European Food and Safety Authority; GCA: Gene conversion assay; MN: Micronucleus test; OECD: Organization for Economic Co-operation and Development; RA: Rec-assay; SCE: Sister chromatid exchange test; SCF: Scientific Committee on Food; TDI: Total daily intake; UL: Tolerable upper intake level. * Regulation repealed by Regulation (EU) 2015/2283. ** A third article EFSA included in the evaluation of the in vitro genotoxicity of taurine was not found in the available literature.

**Table 3:** Results of the miniaturized Ames test for betaine, choline, and taurine in *S. typhimurium* TA1535, TA97a, TA98, TA100, and TA102 with (S9+) and without (S9−) external metabolic activation. Mean ± SD revertants/well of the technical triplicates in an experiment are shown. Positive controls (C+) are shown as mean ± SD revertant/well of all the independent experiments.

Concentration (µg/well)	Revertants/Well									
	TA1535		TA97a		TA98		TA100		TA102	
	S9−	S9+	S9−	S9+	S9−	S9+	S9−	S9+	S9−	S9+
Betaine										
0	17 ± 12	3 ± 1	36 ± 7	56 ± 2	5 ± 3	9 ± 0	42 ± 8	44 ± 3	78 ± 13	66 ± 9
21	2 ± 0	4 ± 2	45 ± 3	68 ± 1	6 ± 4	7 ± 0	34 ± 4	44 ± 8	92 ± 19	86 ± 13
62	2 ± 1	5 ± 1	37 ± 5	63 ± 7	4 ± 2	tox	38 ± 12	42 ± 7	94 ± 13	72 ± 15
185	1 ± 1	2 ± 2	37 ± 6	56 ± 4	4 ± 1	tox	33 ± 8	46 ± 4	86 ± 19	77 ± 18
556	3 ± 2	3 ± 2	40 ± 5	57 ± 5	4 ± 1	tox	45 ± 5	37 ± 4	89 ± 23	67 ± 6
1667	3 ± 1	4 ± 1	35 ± 3	54 ± 7	5 ± 3	tox	43 ± 6	41 ± 5	106 ± 20	85 ± 14
5000	3 ± 0	3 ± 1	39 ± 3	60 ± 7	3 ± 1	tox	45 ± 3	44 ± 1	82 ± 12	83 ± 36
Choline										
0	10 ± 2	10 ± 3	47 ± 1	64 ± 15	8 ± 2	9 ± 3	51 ± 4	50 ± 7	111 ± 20	117 ± 8
21	10 ± 2	10 ± 3	49 ± 6	72 ± 9	6 ± 1	9 ± 4	42 ± 3	47 ± 10	88 ± 26	77 ± 2
62	10 ± 2	8 ± 2	43 ± 5	65 ± 8	7 ± 1	9 ± 4	36 ± 8	40 ± 6	101 ± 16	86 ± 12
185	6 ± 5	8 ± 4	42 ± 7	68 ± 6	7 ± 3	9 ± 1	39 ± 7	51 ± 5	96 ± 30	108 ± 22
556	8 ± 3	7 ± 2	48 ± 3	71 ± 14	6 ± 1	6 ± 1	44 ± 5	50 ± 6	106 ± 13	107 ± 14
1667	6 ± 1	7 ± 3	43 ± 12	66 ± 6	7 ± 3	5 ± 3	36 ± 10	52 ± 6	99 ± 12	95 ± 24
5000	10 ± 4	8 ± 2	45 ± 4	66 ± 6	5 ± 1	8 ± 2	37 ± 9	48 ± 5	98 ± 13	89 ± 24
Taurine										
0	13 ± 1	9 ± 2	34 ± 5	59 ± 5	6 ± 4	9 ± 5	37 ± 1	42 ± 5	119 ± 13	152 ± 14
7	11 ± 4	9 ± 0	34 ± 5	45 ± 8	7 ± 3	5 ± 1	32 ± 2	33 ± 12	111 ± 7	135 ± 4
21	8 ± 2	6 ± 1	30 ± 5	42 ± 7	8 ± 1	10 ± 2	31 ± 1	41 ± 5	110 ± 7	130 ± 6
62	13 ± 2	13 ± 2	43 ± 6	52 ± 7	6 ± 2	8 ± 1	35 ± 7	40 ± 6	134 ± 15	139 ± 4
185	9 ± 1	11 ± 2	40 ± 10	50 ± 13	4 ± 2	11 ± 1	33 ± 3	44 ± 2	114 ± 17	114 ± 4
556	6 ± 3	7 ± 2	34 ± 2	48 ± 7	7 ± 1	9 ± 3	34 ± 9	45 ± 2	115 ± 8	119 ± 7
1667	5 ± 1	9 ± 4	33 ± 6	43 ± 5	7 ± 1	8 ± 2	33 ± 4	35 ± 2	126 ± 37	126 ± 9
C+	145 ± 107	92 ± 11	248 ± 44	460 ± 40	375 ± 60	213 ± 149	225 ± 19	358 ± 34	262 ± 58	153 ± 17

Note. tox: toxicity observed. Positive controls: without S9: 2 μg/well AA for TA97a and TA1535, 10 μg/well AF for TA98 and TA100, and 20 μg/well AF for TA102; with S9: 10 μg/well NPD for TA97a, 20 µg/well NPD for TA98 and TA100, 0.625 μg/well MitC C for TA102, and 2 μg/well NAAZ for TA1535.

**Table 4:** Micronucleus (MN) test in TK6 cells after 3 h treatment with betaine, choline, and taurine with (S9+) and without (S9−) metabolic activation, and 24 h without metabolic activation. MN per 10^3^ nucleated cells and relative survival rate (RS) of the two independent experiments are shown, together with mean ± min/max values. Positive controls (CP, COL) are shown as mean ± SD of MN/10^3^ cells for all the independent experiments.

Treatment (µg/mL)			Experiment 1		Experiment 2		Mean ± min/max			
			MN/10^3^ c.	RS	MN/10^3^ c.	RS	MN/10^3^ c.		RS	
Betaine	3 h (S9−)	0	2.8	100	3.4	100	3.1	±0.3	100	±0
		74	3.5	96	3.9	95	3.7	±0.2	96	±0
		222	6.9	107	5.8	93	6.4	±0.6	100	±7
		667	4.5	103	5.7	100	5.1	±0.6	102	±2
		2000	4.9	103	3.8	96	4.3	±0.5	99	±4
	3 h (S9+)	0	3.0	86	4.7	130	3.9	± 0.9	108	± 22
		74	2.4	67	3.3	117	2.9	±0.5	92	±25
		222	4.0	86	7.6	142	5.8	±1.8	114	±28
		667	6.9	90	5.5	130	6.2	±0.7	110	±20
		2000	5.7	100	8.3	91	7.0	±1.3	96	±5
	24 h (S9−)	0	3.3	100	5.5	100	4.4	±1.1	100	±0
		74	5.0	58	4.5	99	4.8	±0.3	79	±21
		222	3.6	47	3.6	87	3.6	±0.0	67	±20
		667	5.6	52	2.7	81	4.2	±1.5	67	±15
		2000	7.3	59	8.3	81	7.8	±0.5	70	±11
Choline	3 h (S9−)	0	2.8	100	3.5	100	3.2	±0.4	100	±0
		74	3.7	100	3.1	109	3.4	±0.3	104	±4
		222	9.1	95	2.4	150	5.8	±3.3	122	±27
		667	6.7	119	2.8	142	4.7	±1.9	130	±11
		2000	5.3	97	6.6	110	6.0	±0.7	103	±6
	3 h (S9+)	0	4.7	160	5.8	132	5.3	±0.6	146	±14
		74	6.8	102	4.5	105	5.7	±1.2	104	±2
		222	5.2	93	7.2	110	6.2	±1.0	102	±9
		667	4.5	106	8.8	140	6.7	±2.2	123	±17
		2000	4.8	104	4.6	103	4.7	±0.1	104	±1
	24 h (S9−)	0	2.5	100	3.2	100	2.9	±0.4	100	±0
		74	2.7	85	3.6	107	3.2	±0.5	96	±11
		222	2.8	84	5.1	98	4.0	±1.2	91	±7
		667	3.9	68	4.6	95	4.3	±0.4	82	±14
		2000	3.5	80	6.0	92	4.8	±1.3	86	±6
Taurine	3 h (S9−)	0	7.2	100	7.4	100	7.3	±0.1	100	±0
		74	1.9	117	9.4	81	5.7	±3.8	99	±18
		222	3.1	104	5.9	104	4.5	±1.4	104	±0
		667	2.1	102	9.2	107	5.7	±3.6	105	±3
		2000	3.3	114	6.8	92	5.1	±1.8	103	±11
	3 h (S9+)	0	5.6	81	14.3	125	10.0	±4.4	103	±22
		74	5.0	119	11.9	110	8.5	±3.5	115	±5
		222	4.2	99	6.5	106	5.4	±1.2	103	±4
		667	3.4	98	10.4	116	6.9	±3.5	107	±9
		2000	6.1	76	11.2	96	8.7	±2.6	86	±10
	24 h (S9−)	0	5.2	100	2.7	100	4.0	±1.3	100	±0
		74	6.4	116	4.1	81	5.3	±1.2	99	±17
		222	6.3	83	4.0	75	5.2	±1.1	79	±4
		667	10.1	76	6.1	64	8.1	±2.0	70	±6
		2000	6.1	65	3.6	74	4.9	±1.3	70	±5
CP (S9−)							6.1	±2.5		
CP (S9+)							38.1	±22.3		
COL							89.1	±42.4		
